# Intracavity Measurement Sensitivity Enhancement without Runaway Noise

**DOI:** 10.3390/s21248473

**Published:** 2021-12-19

**Authors:** Luke Horstman, Jean-Claude Diels

**Affiliations:** 1 School of Optical Science and Engineering, University of New Mexico, Albuquerque, NM 87106, USA; ljh48332@unm.edu; 2 Center for High Technology Materials, University of New Mexico, Albuquerque, NM 87106, USA; 3 Department of Physics and Astronomy, University of New Mexico, Albuquerque, NM 87106, USA

**Keywords:** intracavity phase interferometry, laser sensors, sensitivity enhancement, precision sensing, inertial sensors, petermann excess noise factor, gyroscopes, ultrafast

## Abstract

A method to increase the sensitivity of an intracavity differential phase measurement that is not made irrelevant by a larger increase of noise is explored. By introducing a phase velocity feedback by way of a resonant dispersive element in an active sensor in which two ultrashort pulses circulate, it is shown that the measurement sensitivity is elevated without significantly increasing the Petermann excess noise factor. This enhancement technique has considerable implications for any optical phase based measurement; from gyroscopes and accelerometers to magnetometers and optical index measurements. Here we describe the enhancement method in the context of past dispersion enhancement studies including the recent work surrounding non-Hermitian quantum mechanics, justify the method with a theoretical framework (including numerical simulations), and propose practical applications.

## 1. Introduction

As the world strives for more adventurous space missions, higher precision studies of fundamental physics and sleek wearable technology, the need for compact and energy efficient, but highly sensitive, measurement devices has never been greater. Interferometry has been a tool for precision measurements since long before the invention of the laser [1,2,3,4]. It involves comparing two paths (sample and reference) of single frequency or multi-color light. The result of the phase measurement is generally observed as an amplitude modulation of passively interfering beams [5], or as a beat frequency in the case of an active interferometer such as the laser gyroscope [6]. Implementing the latter with frequency combs instead of continuous wave (cw) lasers has been termed, “Intracavity Phase Interferometry” (IPI), and promises a wide range of sensing applications [7].

With few exceptions [7,8,9] most reports based on intracavity phase sensing have been limited to cw lasers, i.e., the gyroscope. In this context, a recent hot topic is a byproduct of the field of non-Hermitian quantum mechanics and the discovery of the Exceptional Point (EP) [10]. By placing a sensor at a specific location in parameter space (the EP), it has been shown that some devices can exhibit significantly increased sensitivity [11,12]. Unfortunately, these enhancements generally come at the expense of increased noise [13,14]. For the specific case of the laser gyroscope, the EP is equivalent to the dead band edge where noise and instability dominate [13,14,15,16,17].

Tailoring cavity dispersion to increase detector sensitivity has been an active area of study in the field of laser sensing [18,19]. Though, it has been shown that “slow light” media causes a reduction in sensitivity to resonator path length changes, while “fast light” sensors result in a linewidth broadening that exactly cancels the added sensitivity benefit [20,21,22]. Recently, the critical anomalous dispersion point spoken about in “fast light” lasers, and their corresponding parasitic noise, have been equated to the EP of non-Hermitian quantum mechanics [13,16,17]. By equating the two areas of study, the experimental work regarding the critical anomalous dispersion point can be reexamined in the context of EP theory. One can then conclude that the broadened linewidth is caused by coupling between modes as quantified by the Petermann excess noise factor. This multiplicative factor to the Schawlow-Townes linewidth describes a linewidth broadening that is generated by mode non-orthagonality resulting from mode coupling [23]. Since most, if not all, previous studies in this field have been in reference to cw lasers, the claim of this paper is that by reducing the coupling between the modes by way of an ultrafast intracavity phase interferometry sensor [7], the linewidth broadening typically encountered by dispersion enhancement can be avoided.

### 1.1. Intracavity Phase Interferometry

Most laser based sensors include a laser source separate from the sensing element. This independent sensing element is frequently inserted in a resonator, which increases the sensitivity based on its quality, “*Q*”, factor. A physical quantity (i.e., a stress in a waveguide, a change in molecular state in a high quality Fabry-Perot, etc…) that changes the phase inside the resonator is monitored by a change in transmission. The sensitivity to phase change is multiplied by *Q* because of the repeated passage through the phase perturbation. These detectors typically use a cw laser source with utmost stretching of the coherence time leading to a precisely defined single frequency. The opposite extreme is to use ultrashort pulses. Ultrafast lasers include a mode-locking mechanism that generates a train of equally spaced ultrashort pulses of which the spectrum is a frequency comb. The width of each tooth of the comb can be as narrow as that of the best cw lasers [24,25,26]. Inside the mode-locked laser, a pulse makes a round-trip in a time, $\tau_{rt}$, at a corresponding average group velocity which defines the spacing of the comb teeth as $1/(2\pi \tau_{rt})$. This is not to be confused with the average phase round-trip time, $\tau_{p}$, which is associated with the empty cavity resonance frequency. It has been demonstrated experimentally [27,28] and theoretically [29] that the tooth spacing within a comb is rigorously constant. The frequency comb extends over a large bandwidth such that only some of the central teeth of the comb have their frequency at exact resonance with the empty cavity (or with some intracavity tuning element [30,31]). The first tooth of the extended comb at the frequency $f_{0}$ is called the “carrier to envelope offset frequency” (CEO). This latter quantity is extremely sensitive to any phase perturbation inside the laser cavity. The frequency $\nu_{m}$ of a tooth *m* of the comb is defined as:

(1) $$\nu_{m}=f_{0}+m\frac{1}{2\pi \tau_{rt}}.$$

Rather than having a separate laser source and measuring interferometer, both subject to their own uncorrelated fluctuations and noise, the type of sensing technique analyzed here, termed “Intracavity Phase Interferometry” or IPI, has the laser source and measuring interferometer as a single unit. Two pulses, instead of one, are made to circulate in the laser cavity; one pulse serves as “reference” while the phase of the other is affected at each round-trip by the parameter to be measured. Dual correlated frequency combs are produced by forcing the two pulses to have the same group velocity in the same mode-locked cavity. This source becomes an ultra-sensitive sensor if the physical quantity to be measured causes a differential phase shift Δφ per round-trip between the two pulses. This differential phase shift causes a mode splitting, $\delta_{0}=\Delta \varphi /\left(2\pi \tau_{p}\right)=f_{02}-f_{01}$, where $f_{02}$ and $f_{01}$ are the CEO frequencies defined in Equation (1) for the two circulating pulses. For an ideal source where no linear or nonlinear coupling exists between the two circulating pulses, there will be a frequency split $\Delta \nu =\delta_{0}$ between the two generated frequency combs. In an imperfect world where a phase coupling exists between the two circulating pulses, $\Delta \nu \Rightarrow \Delta \nu \left(\delta_{0}\right)\neq \delta_{0}$. Since the two frequency combs emitted by the source have the same repetition rate, interfering them on a detector produces a beat note of frequency Δν. We define the slope sensitivity of the device as,

(2) $$S=\frac{d\Delta \nu }{d\delta_{0}}.$$

This is not to be confused with the “scale factor”, *S*, used by the gyroscope community. The scale factor relates the rotation rate to the mode-splitting, $\delta_{0}=S\Omega_{R}$. Instead the slope sensitivity defined by Equation (2) is general in the sense that it relates the mode-splitting to the beat frequency read out by the device, $\Delta \nu =S(\delta_{0})$.

The method described here differs from standard dual comb spectroscopy [32,33] in several ways. First, the teeth of the two combs being equal (and equally spaced) implies that all the teeth of the comb contribute to a beat signal with 100% modulation. Because the two combs are correlated, the bandwidth of the beat note signal at Δν is typically less than 1 Hz [34] which is considerably narrower than the bandwidth of an individual tooth of either comb (That bandwidth is typically > 10 MHz in all the measurements performed so far with unstabilized lasers). Another distinction is that recent experimental data did not show a measurable beat note bandwidth dependence on $\delta_{0}$ [35,36], in agreement with simulations presented in Section 5. The phase measurement takes place at each round-trip and, hence, at the cavity round-trip frequency f>100 MHz. Therefore, the 1/f classical noise is minimized in the beat note measurement.

**Figure 1:**
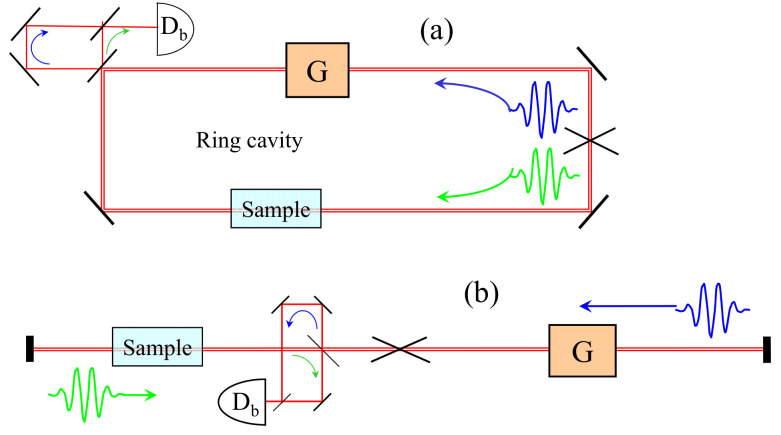
Idealized design of an intracavity phase interferometer, in a ring (**a**) or linear (**b**) configuration. Two pulses (green and blue) are circulated in the cavity. Sample is a device that produces, at each round-trip, a differential phase shift between the two pulses that is proportional to the quantity to be measured. This phase difference results in an optical frequency difference. The latter is measured as a beat note between the two output combs made to interfere via an optical delay line on a detector $D_{b}$. *G* is the gain element.

Figure 1 shows two examples of sensor configurations, where “sample” stands for any phenomenon that can produce a differential phase shift between the two pulses.

The first implementation of a laser cavity with mode-locked pulses propagating in opposite directions utilized a moving saturable absorber. In this configuration of “colliding pulse mode-locking”, the pulses meet in the saturable absorber. Because of the motion of the saturable medium, a dead band is averted though the randomization of the backscattered phase [37]. The first application of the configuration of Figure 1a was to measure air flow through the Fresnel drag [38]. The gyroscope response and the absence of dead band were confirmed in [39]. A ring laser configuration applied to the determination of the Verdet constant was demonstrated in [40]. The magnetic field induced phase measurement was shown to have a 1000 times better signal to noise than a Faraday rotation measurement. This is because, unlike the traditional measurement of Faraday rotation, the measurement of a beat frequency is independent of the amplitude of the laser, and hence is not affected by amplitude noise. A phase shift corresponding to a Faraday rotation of 10${}^{-9}$ radian was easily measured. An atomic magnetometer [41] employing IPI could be used to reduce the large apparatuses required for brain scans in magnetoencephalography that typically rely on superconductors [42]. The linear configuration of Figure 1b was first applied to the measurement of an electro-optic coefficient [43]. It was next realized that an Optical Parametric Oscillator (OPO) configuration offered better signal to noise, leading to a beat frequency bandwidth of 0.17 Hz [34], or a phase resolution of 1.5 $\times 10^{-8}$ radian. This linear configuration was applied to the measurement of a nonlinear index [34]. Another sensing application has been scatterometry. Back-scattering coefficients as small 10${}^{-15}$ can be resolved in transparent media [44] or on reflecting surfaces [45]. IPI has been applied to the study of nonlinear dispersion associated with a three-level Λ system in atomic vapors [46].

These applications, summarized in a review article [7], involved discrete component solid state lasers. Recent trends are to apply these techniques to fiber lasers, with a considerable advantage in reduced size and energy consumption [47]. In the fiber implementation of the linear configuration of Figure 1b the “green” and “blue” pulses are orthogonally polarized, and the “sample” is a Michelson type interferometer with a polarizing beam splitter separating pulses in two branches. IPI is now an accurate monitor of submicron differences in optical path. The device can be used as an accelerometer if one branch is terminated by an inertial mass. Pressure sensing is an active area of study where IPI has yet to be applied. Instead of a piezoresistive based sensor, such as in [48], the linear configuration of IPI could be used, where one pulse is reflected off the wall of a vessel. While not providing a direct electrical response as in [48], it may find a niche of application for its extreme sensitivity and non-contact reading.

If *ℓ* is the length of the sample element inside the laser resonator, the two light beams will experience at each passage through the cavity (round-trip time at the phase velocity $\tau_{p}$) a phase difference Δφ=Δ(kℓ), (wave vector k=2πn/λ where λ is the wavelength in vacuum and *n* the index of refraction) resulting in a differential mode-shift:

(3) $$\delta_{0}=\frac{\Delta \varphi }{2\pi \tau_{p}}=\frac{\Delta (k\ell )}{2\pi \tau_{p}}=\nu \frac{\Delta P}{P}.$$

The last term in Equation (3) expresses that the differential mode shift is the product of the change in cavity perimeter ΔP relative to the total cavity perimeter *P* by the optical frequency ν. It also shows that, in general, $\delta_{0}$ is inversely proportional to the linear dimensions and, therefore, miniaturization is highly desirable. The exception is precisely the most studied case of the laser gyroscope, where the response to a rotation rate $\Omega_{R}$ (s${}^{-1}$) is,

(4) $$\delta_{0}=\frac{4A}{P\lambda }\Omega_{R},$$

proportional to the ratio of the area (*A*) over the perimeter (*P*) and, hence, proportional to the linear dimensions.

The gyro Equation (4) derives directly from the more general detector response (3) where, for a circular cavity of radius *R*, the rotation induces a difference in optical path between counter-circulating pulses of $\Delta P=2R\Omega_{R}\tau_{p}$. The fact that $\Delta P=2R\Omega_{R}P/c$ is proportional to the square of the linear dimensions explains that the rotation response is proportional to the linear dimension. This is in contrast to all other sensors based on IPI. The rotation response (4) being the Sagnac phase shift $\Delta \varphi_{s}$ divided by $2\pi \tau_{p}$ leads to:

(5) $$\Delta \varphi_{s}=\frac{8\pi A}{\lambda }.$$

This is the Sagnac phase shift of which the direct derivation requires special relativity [49,50]. Using the Lorentz transformation, one finds that the time difference for completing a loop by two counter-circulating light beams is $\Delta \varphi_{s}/\omega =\left(2/c^{2}\right)\oint v.d\ell =\left(2/c^{2}\right)\oint \vec{\Omega_{R}}\times \vec{d\ell }.\vec{d\ell }=\left(4/c^{2}\right)\oint \Omega_{R}dA=\left(4/c^{2}\right)\Omega_{R}A$.

The ring is the only topology possible with a cw laser where the two beams occupy the same space at all times. In a mode-locked laser, where two intracavity pulses circulate, each pulse occupies a different position in space at a given instant, and the linear equivalent of the laser gyroscope is possible [Figure 1b]. The linear cavity correspondent of the laser gyroscope is an accelerometer where the “sample” is the position of an inertial mass affecting only one of the pulses.

### 1.2. Paper Outline

In order to show that dispersion tailoring enhancement does not lead to additional noise in an IPI sensor, two equivalent simulation models will be used in sequence. In Section 2, two short pulses step through a mode-locked laser cavity one round trip at a time, producing a series of pulses through an output mirror. The resulting pulse trains in time are Fourier transformed to produce two frequency combs. The beat note between these two combs is analyzed and compared to the mode shift $\delta_{0}$. In the presence of coupling, the beat note is smaller than the mode shift, approaching zero at a point defined as the EP in the language of quantum mechanics. The closer the mode shift approaches the EP, the larger the slope of the beat note response versus mode-shift becomes, which has been hailed as an enhancement [11,12,51]. An increase in slope sensitivity S defined in Equation (2) is only relevant if it is not at the expense of increased noise. The Petermann factor [23,52,53,54] is introduced (Section 4) to quantify the noise contribution brought by the enhancing parameter. Calculations of the Petermann factor get very involved in multimode systems. Fortunately, the Petermann factor correlates with the beat note bandwidth, which is a more convenient calculation in the frequency domain, and easily accessed experimentally. Therefore, a second model operating entirely in the frequency domain is introduced in Section 3. The frequency domain model is more convenient to analyze the dispersion enhancement which is presented in Section 5. It is shown that, in the absence of coupling between the two modes, the response to the mode splitting created by the physical quantity to be measured is increased and remains a linear function of the mode splitting. Finally, Section 6 describes how the enhancement technique is applied experimentally, including preliminary experimental results.

## 2. Time Domain Laser Model

We consider picosecond (or femtosecond) pulses represented by their electric field, $E_{1,2}\left(x,t\right)=\frac{1}{2}{\tilde{E}}_{1,2}\left(x,t\right)\exp\left[i\left(\omega t-kz\right)\right]$; *x* being a round-trip index and ω the average angular frequency of the emitted radiation. As mentioned in the introduction, the two pulses interact with different cavity modes (at $\omega /\left(2\pi \right)\pm \delta_{0}/2$) because of the differential phase shift per round-trip between the two pulses caused by the physical quantity to be measured. The mode splitting implies a k-vector splitting $\Delta k=2\pi \delta_{0}/c$. In the slowly varying envelope approximation, the propagation of the two field envelopes is given by:

(6) $$\begin{array}{ccc} \frac{\partial {\tilde{E}}_{1}}{\partial z} & = & \frac{\alpha_{1}}{L}{\tilde{E}}_{1}+i\frac{\Delta k}{2}{\tilde{E}}_{1}+\frac{s}{L}{\tilde{E}}_{2}\\ \frac{\partial {\tilde{E}}_{2}}{\partial z} & = & \frac{\alpha_{2}}{L}{\tilde{E}}_{2}-i\frac{\Delta k}{2}{\tilde{E}}_{2}+\frac{s}{L}{\tilde{E}}_{1}. \end{array}$$

A non-conservative coupling, represented by the complex number, *s*, is assumed between the two modes. This coupling can only exist at the point of the cavity where the two pulses overlap at each round-trip. If x=z/L is the round-trip index, Equation (6) becomes:

(7) $$\begin{array}{ccc} \frac{\partial {\tilde{E}}_{1}}{\partial x} & = & \alpha_{1}{\tilde{E}}_{1}+i\frac{\Delta \varphi }{2}{\tilde{E}}_{1}+s{\tilde{E}}_{2}\\ \frac{\partial {\tilde{E}}_{2}}{\partial x} & = & \alpha_{2}{\tilde{E}}_{2}-i\frac{\Delta \varphi }{2}{\tilde{E}}_{2}+s{\tilde{E}}_{1}. \end{array}$$

We use the notation $\Delta \varphi =\Delta kL=2\pi \delta_{0}L/c=2\pi \delta_{0}\tau_{p}$ for the differential phase shift/round-trip. Note that Equation (7) assumes that the net gain coefficients, $\alpha_{i}$, and the coupling, *s*, are small (<1). For femtosecond and picosecond pulses that are shorter than the energy relaxation time of the gain medium, the net gain is given by (there is only self-saturation to be considered here since the two circulating pulses do not meet in the gain medium),

(8) $$\alpha_{i}=\frac{\alpha_{0}}{1+W_{i}/W_{s}}-\beta_{i},$$

where $\alpha_{0}$ is the small signal gain factor per round-trip, $W_{i}=\left(\int \right|E_{i}{|}^{2}dt)/2\eta$ is the pulse energy, $W_{s}$ is the saturation energy density, η is the characteristic impedance of the medium, and $\beta_{i}$ is the linear loss per round-trip for each field.

Let us assume for simplicity that the gain and losses are in equilibrium such that $\alpha_{i}=0$. The coupled-mode system of equations reduces to:

(9) $$\frac{\partial }{\partial x}\left(\begin{array}{c} {\tilde{E}}_{1}\\ {\tilde{E}}_{2} \end{array}\right)=\left[\begin{array}{cc} -i\Delta \varphi /2 & s\\ s & i\Delta \varphi /2 \end{array}\right]\left(\begin{array}{c} {\tilde{E}}_{1}\\ {\tilde{E}}_{2} \end{array}\right).$$

Postulating the existence of a steady-state solution, one uses the Ansatz: ${\tilde{E}}_{i}=A_{i,j}e^{-i\lambda_{j}x}$ for the complex amplitudes where $A_{i,j}$ are constants, and $\lambda_{j}$ are solutions to the Eigenvalue equation:

(10) $$\left|\begin{array}{cc} -i\Delta \varphi /2+i\lambda & s\\ s & i\Delta \varphi /2+i\lambda \end{array}\right|=0,$$

which leads to the Eigenvalues:

(11) $$\lambda_{\pm }=\pm \frac{\sqrt{\Delta \varphi^{2}-4s^{2}}}{2}.$$

The solutions for the fields ${\tilde{E}}_{1}$ and ${\tilde{E}}_{2}$ are a linear combination of the Eigenfunctions. In this particular case of coupling between identical resonators, the beat note frequency is simply $\Delta \nu =\left(\lambda_{+}-\lambda_{-}\right)/\left(2\pi \tau_{p}\right)$. The dead band edge and EP then occur at, $\Delta \varphi^{2}=4s^{2}$.

**Figure 2:**
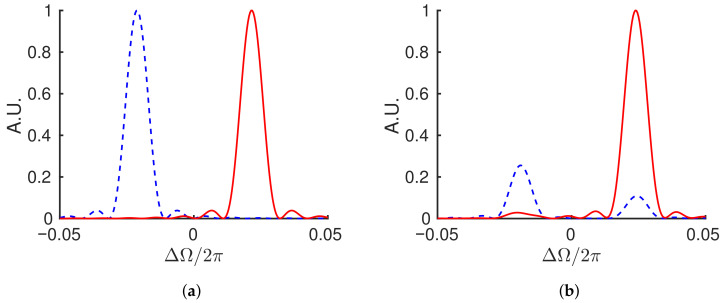
Numerical solution showing a pair of teeth of the two frequency combs (red, blue) defined by the time model of Equation (7) after 100 round-trips with no coupling, s=0 (**a**), and coupling, $s=0.1e^{\left(1i\right)}$ (**b**). Both simulations were carried out with a detuning of Δφ=0.27, gain of $\alpha_{0}=1$, $\beta_{1,2}=0.05$, and $W_{s}=1$ in Equation (8), and $\tau_{p}=1$. When coupling is included, the fields pass energy back and forth such that which field is dominant in plot (**b**) depends on how many round-trips are considered If taken over an infinite number of round-trips, (**b**) and Figure 4b would be symmetric, since the amplitudes of the two pulses follow similar oscillation, but out of phase. Since the variable *x* is in fact the time (in unit of round-trip), (**b**) and Figure 4b can be recognized as wavelet transforms.

Figure 2 shows a pair of teeth of the two frequency combs corresponding to the fields numerically solved from Equation (7). The system of Equation (9) are first solved for in the time domain and then the Fourier transform of the resultant pulse train is taken to obtain the frequency comb. The angular frequency variable, ΔΩ=Ω−ω, is the departure from an average optical angular frequency, ω, such that the differential phase causes a splitting centered at ΔΩ=0. The simulation used a detuning of Δφ=0.27, gain of $\alpha_{0}=1$, $\beta_{1,2}=0.05$, and $W_{s}=1$ in Equation (8), and the temporal dimensions are scaled by the round-trip time ($\tau_{p}=1$). Figure 2a shows the result without coupling, s=0, while Figure 2b shows what happens when a coupling of $s=0.1e^{\left(1i\right)}$ is included. Since $\tau_{p}=1$, the peaks are spaced by $\Delta \nu =\delta_{0}=\varphi /2\pi =0.043$ in frequency space. However, when the electric fields are interfered, the system with coupling will read out a distorted beat frequency that is not necessarily equal to the mode spacing.

### Gain Difference Exceptional Point

As pointed out in the “Gain Difference Exceptional Point” section of [16], the situation described above is slightly different from the case of asymmetric gain considered in [51]. In that case, the two electric fields have the same frequency, and there is not a beat note, but a shift in absolute frequency that depends on the detuning. This is the situation where there is a different gain $\alpha_{1}$ and $\alpha_{2}$ in each of the coupled resonators, and the coupling is purely conservative (*s* = 0 replaced by $\kappa_{1}=\kappa =-\kappa_{2}^{*}$ [16]). The Eigenvalue equation is then:

(12) $$\left|\begin{array}{cc} \alpha_{1}-i\Delta \varphi /2+i\lambda & \kappa \\ -\kappa^{*} & \alpha_{2}+i\Delta \varphi /2+i\lambda \end{array}\right|=0,$$

which leads to the Eigenvalues:

(13) $$\lambda_{\pm }=i\frac{\alpha_{1}+\alpha_{2}}{2}\pm \frac{\chi }{2},$$

with,

(14) $$\chi =\sqrt{{4|\kappa |}^{2}-{\left[(\alpha_{1}-\alpha_{2})-i\Delta \varphi \right]}^{2}}.$$

One can then see that at zero detuning (Δφ=0) the modes collapse to an Exceptional Point when ${4|\kappa |}^{2}={\left(\alpha_{1}-\alpha_{2}\right)}^{2}$. Numerical solutions were presented in [16] showing that the two fields ${\tilde{E}}_{1}$ and ${\tilde{E}}_{2}$ have the same frequency such that no “beat note” can be observed. An analytical expression for the fields ${\tilde{E}}_{1}$ and ${\tilde{E}}_{2}$ is given by:

(15) $$\begin{array}{c} \begin{array}{cc} {\tilde{E}}_{1} & =A_{11}e^{-i\lambda_{+}x}+A_{12}e^{-i\lambda_{-}x}\\ {\tilde{E}}_{2} & =A_{21}e^{-i\lambda_{+}x}+A_{22}e^{-i\lambda_{-}x}, \end{array} \end{array}$$

where the Eigenvalues $\lambda_{\pm }$ are from Equation (13). Choosing $\alpha_{1}=\alpha =\kappa$ and $\alpha_{2}=-\alpha$, the Eigenvalues become,

(16) $$\lambda_{\pm }=\pm \frac{\chi }{2}=\pm \frac{1}{2}\sqrt{-2i\kappa \Delta \varphi -\Delta \varphi^{2}}\approx \pm \sqrt{\frac{\kappa \Delta \varphi }{2}}\sqrt{-i}=\pm \frac{1}{2}\sqrt{\kappa \Delta \varphi }\left(1-i\right),$$

where the approximation is valid since near the EP Δφ is small. By substituting this into Equation (15), it is clear that the imaginary part of $\lambda_{+}$ will cause the first term to decay to zero, while the imaginary part of $\lambda_{-}$ will cause the second term to grow exponentially. Saturation of the gain will stabilize the second term to a finite value, while the first term decays to zero. Therefore, the fields ${\tilde{E}}_{1}$ and ${\tilde{E}}_{2}$ oscillate at the same frequency (dividing by the round-trip factor $2\pi \tau_{p}$) of $\frac{1}{4\pi \tau_{p}}\sqrt{\kappa \Delta \varphi }$, and there is no beat note to be observed. Details of the solution are given in Appendix A.

**Figure 3:**
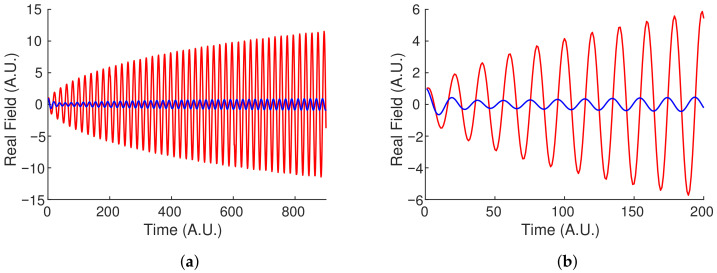
Evolution of the two fields (red, blue) near the “Gain Difference Exceptional Point”. Equation (7) was solved using initial cw fields of amplitude 1 and κ=0.05, a saturable gain with $\alpha_{0}=0.1$, $W_{s}=1$, $\beta_{1}=0$, $\alpha_{2}=-\kappa$ in Equation (8), and Δφ=2π∗0.1. (**b**) is a zoomed in plot of (**a**) showing that the two fields have the same optical frequency, and therefore there is no measureable beat frequency when the two fields are interfered.

The fact that there should be no beat note in this model can be verified by solving numerically the system of coupled differential equations, Equation (12), for a conservative coupling κ=0.05, saturable gain using $\alpha_{0}=0.1$, $W_{s}=1$, $\beta_{1}=0$, $\alpha_{2}=-\kappa$, and Δφ=2π∗0.1. The results are presented in Figure 3 which shows the two fields oscillating with the same optical frequency. Initial cw fields of amplitude 1 were used, and the real fields are plotted as a function of time. Because the real fields are plotted, the oscillations correspond to the optical frequency and thus not directly experimentally accessible like a true beat frequency. Assuming a cavity $\tau_{p}=1$ ns, the displayed period is 20 ns which corresponds to a frequency of 0.05 GHz. This does not agree with the predicted frequency of $\frac{1}{4\pi \tau_{p}}\sqrt{\kappa \Delta \varphi }=0.014$ GHz because, as stated in [16], the relationship $\alpha_{1}-\alpha_{2}=2\kappa$ is not maintained for Δφ>0. In this case the gain in the first resonator saturates to $\alpha_{1}=0.00068$, while the second resonator maintains the constant $\alpha_{2}=-\kappa$. Plugging these values into Equation (13) and taking the real part (since the imaginary part leads to gain or loss) results in the expected value of, $Re\left(\lambda_{\pm }\right)/\left(2\pi \tau_{p}\right)=0.05$.

## 3. Frequency Domain Model

In continuously pumped lasers the noise is due to spontaneous emission from the active medium. The evolution from noise to a regular train of pulses has been the object of numerous theories and computer simulations since the first mode-locked laser was operated (see, for instance, [55]). Instead of using the standard description for frequency combs [56], we proceed in the frequency domain by following an initially broad spectrum through successive round-trips. A pulse evolves in the cavity, and through its round-trip summation a frequency comb develops. Instead of including all nonlinear effects that force the longitudinal modes to be in phase, we assume at the onset that all frequency components are locked together. In other words, it is assumed that the pulse already exists in time and we are simply summing the round-trips. The dual correlated frequency comb is generated by duplicating this evolution in the same cavity (same average round-trip time at the group velocity) but with each pulse having a phase shift of ±Δφ/2, corresponding to the modes being shifted by $\pm \delta_{0}/2$. The advantage of this approach is that the mode linewidth can be controlled by the number of round-trips. We start with a spectrally broad electric field $E_{p,0}\left(\Omega \right)={\tilde{E}}_{p,0}\left(\Delta \Omega \right)\exp\left(-ikz\right)$ where the index p=± refers to each of the two frequency combs, and the cavity is at resonance at the frequency ω (corresponding to ΔΩ=0). The resonance condition is $\omega \tau_{p}=2N\pi$ where *N* is an integer and $\tau_{p}$ is the round-trip time at the phase velocity corresponding to the resonant frequency ω. The spectral field of each circulating pulse at the round-trip x+1 is related to the field at the previous round-trip, *x* by:

(17) $${\tilde{A}}_{\pm ,x+1}={\tilde{A}}_{\pm ,x}e^{-i\tau_{p}\left(\pm \Delta \varphi /2+\Delta \Omega \right)}.$$

where the amplitude notation has been added to distinguish the mathematical round-trip quantity ($\tilde{A}$) from the measurable resultant sum ($\tilde{E}$). The measurable field is the sum after *M* round-trips:

(18) $${\tilde{E}}_{\pm ,M}={\tilde{A}}_{\pm ,0}\sum_{x=0}^{M}e^{-ix\tau_{p}\left(\pm \Delta \varphi /2+\Delta \Omega \right)}.$$

The sum of this geometric series leads to an analytic expression for the two frequency combs:

(19) $${\tilde{E}}_{\pm ,M}={\tilde{A}}_{\pm ,0}\frac{1-{\left[e^{-i\tau_{p}\left(\pm \Delta \varphi /2+\Delta \Omega \right)}\right]}^{M+1}}{1-e^{-i\tau_{p}\left(\pm \Delta \varphi /2+\Delta \Omega \right)}}.$$

The model can be extended to include coupling between the counter-propagating pulses. Since Fourier transformations are linear operations, the coupling can be simply introduced the same way as in the time domain. We choose here the non-conservative coupling *s* introduced in Section 2. With such a coupling an analytical solution such as in Equation (19) is not available, and a numerical method must be sought. A split method is used to create the summation. First, each electric field is propagated a round-trip individually without coupling according to its transfer function $\exp-i\tau_{p}\left(\pm \Delta \varphi /2+\Delta \Omega \right)$. Next, the coupling is applied by adding a proportional amount, *s*, of the opposite propagated field into each other, leading to a coupled amplitude $\tilde{A}c_{\pm ,x}$ at round-trip *x*. The coupling being non-conservative means the total energy will diverge unless a saturable gain is applied to the coupled amplitudes at each round-trip:

(20) $${\tilde{A}}_{\pm ,x+1}=\tilde{A}c_{\pm ,x}+\alpha_{1,2}\tilde{A}c_{\pm ,x}.$$

The resultant comb is again obtained by the summing the round-trips:

(21) $${\tilde{E}}_{\pm ,M}=\sum_{x=0}^{M}{\tilde{A}}_{\pm ,x}.$$

**Figure 4:**
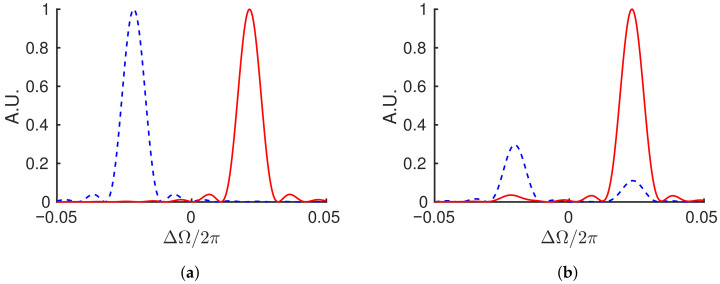
Numerical solution showing a pair of teeth of the two frequency combs (red, blue) defined by Equation (21) after 100 round-trips with no coupling, s=0 (**a**), and coupling, $s=0.1e^{\left(1i\right)}$ (**b**). Both simulations were carried out with a detuning of Δφ=0.27, gain of $\alpha_{0}=1$, β=0.05, and $W_{s}=1$ in Equation (8), and $\tau_{p}=1$. When coupling is included, the fields pass energy back and forth such that which field is dominant in plot (**b**) depends on how many round-trips are considered.

Figure 4 shows the result of the frequency domain calculation using the same parameters as the time domain system displayed in Figure 2. Comparison of the two figures demonstrates the equivalence of the two models. The frequency domain calculation allows a more straightforward approach to the dispersion enhancement that will be introduced in Section 5.

## 4. The Petermann Factor

For the situation of non-conservative coupling (s≠0; κ=0) and balanced gain ($\alpha_{1,2}=0$), the enhancement factor S is:

(22) $$S=\frac{d\Delta \nu }{d\delta_{0}}=\frac{d(\lambda_{+}-\lambda_{-})}{d\Delta \varphi }=\frac{\Delta \varphi }{\sqrt{\Delta \varphi^{2}-4s^{2}}}.$$

The enhancement tends to infinity at the edge of the dead band Δφ=2s, and tends asymptotically to unity as Δφ increases away from the exceptional point. As is well known in the optical gyroscope community, there is no advantage in operating close to the dead band where the beat note bandwidth increases [57,58,59]. The Petermann excess noise factor quantifies the excess noise produced next to an exceptional point which explains the inherent noise that exists within the dead band region.

The excess noise theory has been developed for Schrödinger type systems, or more generally, linear systems with corresponding discrete Eigenfunctions and Eigenvalues. This is the case of modes of a cavity. Specifically, it has been shown that the system of equations describing the evolution of the two pulses in a mode-locked cavity is a Schrödinger-type equation of which the split between the Eigenvalues is the beat note Δν [60]. A good representation of the excess noise added to a laser system due to the modes becoming non-orthogonal is the Petermann factor [23].

(23) $$K_{p}=\frac{1}{1-|\langle \epsilon_{1}|\epsilon_{2}\rangle {|}^{2}},$$

where $|\epsilon_{1}\rangle$ and $|\epsilon_{2}\rangle$ are the two eigenmodes of the system normalized to $\langle \epsilon_{1,2}|\epsilon_{1,2}\rangle =1$ [61]. It has been shown [52,53,54] that the factor $K_{p}$, which is a multiplicative factor of the spontaneous emission noise, becomes >1 in a laser system when the modes $\left|\epsilon_{1,2}\right\rangle$ are non-orthogonal. For many systems showing an enhancement at the Exceptional Point, it has been pointed out that the Petermann factor increases [13,14]. For the case of the two-level system described by Equations (9), The Eigenfunction equation corresponding to the Eigenvalues $\lambda_{\pm }$ are:

(24) $$\begin{array}{ccc} 0 & = & \left(-i\Delta \varphi /2\pm i\frac{\sqrt{\Delta \varphi^{2}-4s^{2}}}{2}\right)x_{1}+sx_{2}\\ 0 & = & sx_{1}+\left(i\Delta \varphi /2\pm i\frac{\sqrt{\Delta \varphi^{2}-4s^{2}}}{2}\right)x_{2}. \end{array}$$

Multiplying the first equation by $i\Delta \varphi /2\mp i\frac{\sqrt{\Delta \varphi^{2}-4s^{2}}}{2}$, the second by *s*, and taking the difference of the two equations gives the identity 0=0. The eigenvectors are then deduced from the first equation to be:

(25) $$\epsilon_{\pm }=\mu_{\pm }\left[\begin{array}{c} 2s\\ i\Delta \varphi \pm i\sqrt{\Delta \varphi^{2}-4s^{2}} \end{array}\right],$$

where $\mu_{\pm }$ is the normalization factor. If the system is just outside of the dead band edge such that $\Delta \varphi^{2}>4s^{2}$, then, to ensure that the normalization $\langle \epsilon_{\pm }\epsilon_{\pm }\rangle =1$ holds, we have to add the rather complicated normalization factor of,

(26) $$\mu_{\pm }=\frac{1}{\sqrt{2\left(\Delta \varphi^{2}\pm \Delta \varphi \sqrt{\Delta \varphi^{2}-4s^{2}}\right)}}.$$

This normalization is required for the Petermann factor to take the simple form shown in Equation (23). The inner product is then,

(27) $$\begin{array}{c} \langle \epsilon_{+}|\epsilon_{-}\rangle =\mu_{+}\mu_{-}\left[2s,-i\Delta \varphi -i\sqrt{\Delta \varphi^{2}-4s^{2}}\right]\left[\begin{array}{c} 2s\\ i\Delta \varphi -i\sqrt{\Delta \varphi^{2}-4s^{2}} \end{array}\right]\\ \;=\frac{2s}{\Delta \varphi }. \end{array}$$

Plugging this into Equation (23) shows explicitly that the Petermann factor diverges as the system approaches the dead band edge at Δφ=2s:

(28) $$K_{p}=\frac{1}{1-\frac{4s^{2}}{\Delta \varphi^{2}}}.$$

The claim of this paper is demonstrated theoretically in Equation (28). If the coupling between the modes is eliminated, s=0, then no additional noise is added since $K_{p}=1$.

Unfortunately, there is no clear way to extract the normalized eigenmodes of Equation (25) from the numerical solutions that solve for the fields ${\tilde{E}}_{\pm }$. However, the fact that $K_{p}$ is a multiplicative factor to the Schawlow–Townes linewidth means that its effect shows up as a broadening of the lasing linewidth. Thus, by showing that the beat signal bandwidth is not broadened when dispersion enhancement is added, the theoretical statement above can be numerically confirmed.

The beat signal on the detector $D_{b}$ (Figure 1) in time is given by,

(29) $$D_{b}\left(t\right)=\frac{1}{2\eta }\left[|{\tilde{E}}_{1}{|}^{2}+{\left|{\tilde{E}}_{2}\right|}^{2}+{\tilde{E}}_{1}^{*}{\tilde{E}}_{2}+{\tilde{E}}_{1}{\tilde{E}}_{2}^{*}\right].$$

Taking the Fourier transform of the beat signal, $\mathrm{FT}\left[D_{b}\left(t\right)\right]=D_{b}\left(\Delta \Omega \right)$, and taking advantage of the linearity and cross-correlation properties of the Fourier transform leads to,

(30) $$\begin{array}{c} \begin{array}{cc} D_{b}\left(\Delta \Omega \right)=\frac{1}{2\eta } & |{\tilde{E}}_{1}\left(\Delta \Omega \right)⊗{\tilde{E}}_{1}\left(\Delta \Omega \right)+{\tilde{E}}_{2}\left(\Delta \Omega \right)⊗{\tilde{E}}_{2}\left(\Delta \Omega \right)\ldots \\ & +{\tilde{E}}_{1}\left(\Delta \Omega \right)⊗{\tilde{E}}_{2}\left(\Delta \Omega \right)+{\tilde{E}}_{2}\left(\Delta \Omega \right)⊗{\tilde{E}}_{1}\left(\Delta \Omega \right){|}^{2}. \end{array} \end{array}$$

where the cross-correlation function is defined as,

(31) $$A\left(x\right)⊗B\left(x\right)=\int_{-\infty }^{\infty }A^{*}\left(\tau \right)B\left(x+\tau \right)d\tau .$$

The beat signal bandwidth can then be calculated as the square-root of the mean square deviation (second central moment):

(32) $$\langle {\ddot{\phi }}^{2}\rangle =\frac{\int_{-\infty }^{\infty }{\left(\Delta \Omega -2\pi \Delta \nu \right)}^{2}D_{b}\left(\Delta \Omega \right)d\Delta \Omega }{\int_{-\infty }^{\infty }D_{b}\left(\Delta \Omega \right)d\Delta \Omega }.$$

Equation (32) will be used in the next section to quantify how much noise is added to the enhanced system. The average beat frequency will be calculated as it would be measured experimentally as the center of gravity of the beat signal spectrum,

(33) $$\Delta \nu =\frac{1}{2\pi }\frac{\int_{-\infty }^{\infty }\Delta \Omega D_{b}\left(\Delta \Omega \right)d\Delta \Omega }{\int_{-\infty }^{\infty }D_{b}\left(\Delta \Omega \right)d\Delta \Omega }.$$

## 5. Enhancement through Resonant Dispersion

If an additional dispersive element is inserted into the cavity with a periodic transfer function of $\tilde{h}=e^{-i\psi (\Delta \Omega )}$, then Equation (17) becomes:

(34) $${\tilde{A}}_{\pm ,x+1}={\tilde{A}}_{\pm ,x}e^{-i[\pm \left(\Delta \varphi /2\right)\tau_{p}+\tau_{p}\Delta \Omega +\psi \left(\Delta \Omega \right)]}.$$

Using a Taylor expansion limited to first order $\psi \left(\Delta \Omega \right)\approx \psi_{0}+\Delta \Omega {\left.\partial \psi /\partial \Delta \Omega \right|}_{0}$, and ignoring the global phase factor $\psi_{0}$ since it affects all teeth on each comb equally results in:

(35) $${\tilde{A}}_{\pm ,x+1}={\tilde{A}}_{\pm ,x}e^{-i\left[\pm \left(\Delta \varphi /2\right)\tau_{p}+\left(\tau_{p}+\frac{\partial \psi }{\partial \Delta \Omega }{|}_{0}\right)\Delta \Omega \right]}.$$

By comparing Equation (17) and Equation (35), it is clear that the effect of adding an intracavity dispersive element is to modify the phase round-trip time as $\tau_{p}\Rightarrow \tau_{p}{+\partial \psi /\partial \Delta \Omega |}_{0}$. Inserting this into Equation (3) results in a modified detuning of,

(36) $$\delta =\frac{\Delta \varphi }{2\pi (\tau_{p}+\frac{\partial \psi }{\partial \Delta \Omega }{|}_{0})}=\frac{\frac{\Delta \varphi }{2\pi \tau_{p}}}{1+\frac{1}{\tau_{p}}\frac{\partial \psi }{\partial \Delta \Omega }{|}_{0}}=\frac{\delta_{0}}{1+\frac{1}{\tau_{p}}\frac{\partial \psi }{\partial \Delta \Omega }{|}_{0}}.$$

The enhancement factor defined in Equation (2) now becomes:

(37) $$S=\frac{d\Delta \nu }{d\delta_{0}}=\frac{\partial \Delta \nu }{\partial \delta }\frac{\partial \delta }{\partial \delta_{0}}=S_{1}S_{2}=S_{1}\frac{1}{1+\frac{1}{\tau_{p}}\frac{\partial \psi }{\partial \Delta \Omega }{|}_{0}}.$$

$S_{1}=\Delta \nu /\delta$ is the scale factor enhancement typically reported by the gyroscope community. If $S_{1}$ is enhanced by way of placing the system at the Exceptional Point, it also comes with an increase in excess noise [13,14,16]. $S_{2}=\delta /\delta_{0}$ is a real mode-spacing enhancement (using the method described above) which maintains the linearity of the response in the absence of coupling as long as the modes remain in the linear region of the linear dispersion. If there is negative (anomalous) resonant group-velocity dispersion (${\partial \psi /\partial \Delta \Omega |}_{0}<0$) then S>1, and the mode splitting $\delta =S\delta_{0}$ is enhanced. This situation occurs in the dispersive region of an absorbing resonance. The case of dispersion caused by a gain resonance represents a decrease in sensitivity (S<1).

**Figure 5:**
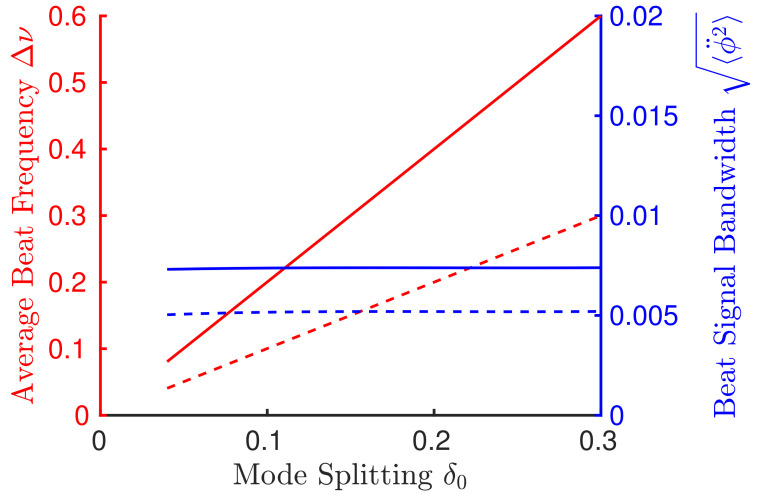
Enhanced (solid) and non-enhanced (dashed) sensor response curves. The average beat frequency (left axis, red) shows Equation (33) plotted as function of the applied mode-splitting. The result of using the enhancement factor of ${\partial \psi /\partial \Delta \Omega |}_{0}=-0.5$ acts to increase the sensitivity response, S (observed as the slope of the average beat frequency curve), without causing the noise to diverge as characterized by the beat signal bandwidth, Equation (32) (right axis, blue). 150 round-trips were used in these calculations, without coupling, and gain parameters of $\alpha_{0}=1$, γ=0.05, and $W_{s}=1$.

Figure 5 shows the enhanced (solid) and non-enhanced (dashed) sensor response curve and beat signal bandwidth. The average beat frequency (red, left axis) is plotted as a function of mode splitting according to Equation (33). The enhancement is clear as the slope of the lines correspond to the enhancement factor S. The beat signal bandwidth (blue, right axis) is plotted according to Equation (32), implying that the noise does not diverge since the coupling has been removed, s=0. This calculation was carried out using $\tau_{p}=1$, with 150 round-trips, gain parameters of $\alpha_{0}=1$, γ=0.05, and $W_{s}=1$, and an enhancement factor of ${\partial \psi /\partial \Delta \Omega |}_{0}=-0.5$.

Not only does this enhancement technique maintain its viability when considering the effect of excess noise, but the dead band is eliminated entirely. This is in contrast to the EP enhancement where the noise can be seen to diverge near the dead band edge. The added instability created by operating the sensor near its dead band edge washes out any perceived sensitivity enhancement, which has been shown both experimentally [14] and theoretically both in this paper and in [13,16].

## 6. Practical Implementation of the Enhancement

**Figure 6:**
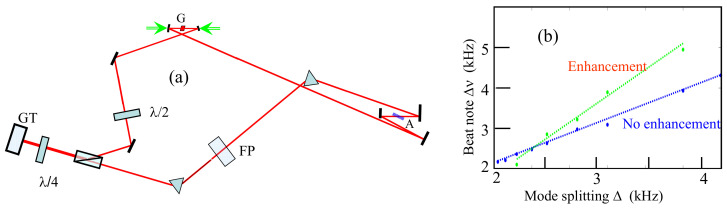
(**a**) Ring cavity with tail: G—gain medium; A—saturable absorber; P—polarizer; FP—uncoated etalon, 15 mm thickness; GT—Gires–Tournois, 15 mm thickness. (**b**) Comparison of the beat note measured without FP and GT, with the beat note response with FP and GT.

It is implied in the previous section that the dispersion enhancement applies to each mode of the frequency comb. Because the teeth of a frequency comb are equally spaced, it is sufficient to lock the modes of a laser to a resonant structure with much larger mode spacing. As demonstrated experimentally in [62], when an etalon (uncoated fused silica plate of 15 mm thickness) is inserted in a mode-locked laser cavity, it pins the comb teeth to the resonant modes of that Fabry–Perot. In addition, as a result of the etalon acquiring the finesse of the laser, its transmission and dispersion are narrow compared with the mode spacing of the laser. Because the dispersion is positive, Equation (37) implies a reduction of S. In order to achieve negative dispersion, the Fabry–Perot has to be used in reflection, i.e., as a Gires–Tournois end mirror. With an intracavity Fabry-Perot, the frequency comb will lock to the transmission resonances automatically because this is a configuration of minimum losses [62]. This is not the case when a Gires–Tournois is implemented, where some stabilization mechanism has to be used to force the modes to coincide. A preliminary demonstration of enhancement shown in Figure 6 was achieved by using an intracavity etalon in transmission to force the modes to coincide, and a Gires–Tournois end mirror of the same thickness and high finesse for enhancement [35]. In the case of a linear cavity, the Gires–Tournois can simply be at an end mirror. In the case of a ring cavity, a Gires–Tournois designed for normal incidence can be the end mirror of a tail [63] as in Figure 6a. The non-enhanced response is compared to the response of the ring cavity with the Fabry–Perot and Gires–Tournois combination in Figure 6b.

## 7. Conclusions

Most phase measurements are traditionally performed by passive interferometry where the relative phase of a reference and a sample beam is determined by the amplitude of the sum of the two fields. The laser gyroscope however is an active interferometer, where the Sagnac phase shift is measured as a beat frequency. By using mode-locked lasers in which two pulses circulate, it is shown that active interferometry can be extended to most phase measurement, yielding a beat frequency equal to the phase shift to be measured divided by the laser round-trip time. We define the slope sensitivity of these active sensors as the derivative of the beat frequency with respect to the imposed phase shift (the quantity to be measured). It is shown that these lasers with two intracavity beams can be modeled as non-Hermitian quantum systems. As such, they exhibit some specific location in parameter space, called exceptional points, where the slope sensitivity. We show that this enhancement is generally at the expense of increased noise, such that the net signal to noise is decreased. This unwanted property is generally associated with an intracavity coupling between the two beams circulating in the cavity. In some cases—such as a dual pulse optical parametric oscillator pumped by a mode-locked laser—this coupling is completely eliminated. These two-pulse lasers are modeled in the time and frequency domain. It is shown that the beat note frequency response can be enhanced by introducing resonant intracavity dispersion. Removing the coupling between the two electric fields to be interfered by using non interacting ultrashort pulses recovers the boost in enhancement by maintaining mode orthogonality. The frequency simulation demonstrates the enhancement with a net increase of signal to noise. Preliminary experimental evidence shows promise for future studies to fully realize the enhancement by implementing a Gires–Tournois interferometer end mirror into IPI lasers.

## Data Availability

The data presented in this study are available on request from the corresponding author. The data are not publicly available due to privacy.

## Abbreviations

The following abbreviations are used in this manuscript:

Q-factor	Quality Factor
cw	Continuous Wave
EP	Exceptional Point
IPI	Intracavity Phase Interferometry
Gyro	Gyroscope
CEO	Carrier-envelope-offset

## Appendix A. Analytic Solution to Conservative Coupling Eigenmodes

The round-trip coupled-mode system of equations for a two-level system with conservative coupling is given by,

(A1) $$\frac{\partial }{\partial x}\left(\begin{array}{c} {\tilde{E}}_{1}\\ {\tilde{E}}_{2} \end{array}\right)=\left[\begin{array}{cc} -i\Delta \varphi /2 & \kappa \\ -\kappa^{*} & i\Delta \varphi /2 \end{array}\right]\left(\begin{array}{c} {\tilde{E}}_{1}\\ {\tilde{E}}_{2} \end{array}\right).$$

The Eigenvalues are found by taking the determinant of the Eigenvalue matrix and solving,

(A2) $$\left|\begin{array}{cc} \alpha_{1}-i\Delta \varphi /2+i\lambda & \kappa \\ -\kappa^{*} & \alpha_{2}+i\Delta \varphi /2+i\lambda \end{array}\right|=0,$$

which leads to the Eigenvalues:

(A3) $$\lambda_{\pm }=i\frac{\alpha_{1}+\alpha_{2}}{2}\pm \frac{\chi }{2},$$

where,

(A4) $$\chi =\sqrt{{4|\kappa |}^{2}-{\left[(\alpha_{1}-\alpha_{2})-i\Delta \varphi \right]}^{2}}.$$

The final solutions are a linear combination of the Eigenvalues and eigenvectors:

(A5) $$\begin{array}{c} \begin{array}{cc} {\tilde{E}}_{1} & =A_{1}\epsilon_{+}^{1}e^{-i\lambda_{+}x}+A_{2}\epsilon_{-}^{1}e^{-i\lambda_{-}x}\\ {\tilde{E}}_{2} & =A_{1}\epsilon_{+}^{2}e^{-i\lambda_{+}x}+A_{2}\epsilon_{-}^{2}e^{-i\lambda_{-}x}, \end{array} \end{array}$$

where the Eigenvalues $\lambda_{\pm }$ are given by Equation (A3). The eigenvectors are calculated to be,

(A6) $$|\epsilon_{\pm }\rangle =\left[\begin{array}{c} \epsilon_{\pm }^{1}\\ \epsilon_{\pm }^{2} \end{array}\right]=\left[\begin{array}{c} 1\\ \frac{\alpha_{2}-\alpha_{1}+i\Delta \varphi \pm i\chi }{2\kappa } \end{array}\right],$$

and the amplitudes, based on the intial conditions $E_{1,2}\left(0\right)$, are given by,

(A7) $$\begin{array}{c} \begin{array}{cc} A_{2} & =\frac{i}{2\chi }\left[2\kappa E_{2}\left(0\right)-\left(\alpha_{2}-\alpha_{1}+i\Delta \varphi +i\chi \right)E_{1}\left(0\right)\right]\\ A_{1} & =E_{1}\left(0\right)-A_{2}. \end{array} \end{array}$$

The gain difference Exceptional Point occurs when the gain difference offsets the conservative coupling such that ${\left(\alpha_{1}-\alpha_{2}-i\Delta \varphi \right)}^{2}=4{\left|\kappa \right|}^{2}$. This can be observed by setting $\alpha_{1}=k$, $\alpha_{2}=-k$ and $E_{1,2}\left(0\right)=1$. The equations then reduce to,

$$\begin{array}{cc} \lambda_{\pm } & =\pm \frac{\chi }{2},\\ \chi & =\sqrt{-i2\Delta \varphi \kappa -\Delta \varphi^{2}},\\ A_{2} & =-\frac{\Delta \varphi +\chi }{2\chi },\\ A_{1} & =1-A_{2},\\ \epsilon_{\pm }^{2} & =\frac{-2\kappa +i\Delta \varphi \pm i\chi }{2\kappa }. \end{array}$$

Since the Exceptional Point occurs at Δφ=0, it can be assumed that Δφ is small, such that,

(A8) $$\begin{array}{c} \begin{array}{cc} \lambda_{\pm } & =\pm \frac{\chi }{2}=\pm \frac{1}{2}\sqrt{-2i\kappa \Delta \varphi -\Delta \varphi^{2}}\\ \; & \approx \pm \sqrt{\frac{\kappa \Delta \varphi }{2}}\sqrt{-i}=\pm \frac{1}{2}\sqrt{\kappa \Delta \varphi }\left(1-i\right). \end{array} \end{array}$$

Plugging this into Equation (A5) leads to,

(A9) $$\begin{array}{c} \begin{array}{cc} {\tilde{E}}_{1} & =A_{1}\epsilon_{+}^{1}e^{ix\sqrt{\kappa \Delta \varphi }/2-x\sqrt{\kappa \Delta \varphi }/2}+A_{2}\epsilon_{-}^{1}e^{ix\sqrt{\kappa \Delta \varphi }/2+x\sqrt{\kappa \Delta \varphi }/2}\\ {\tilde{E}}_{2} & =A_{1}\epsilon_{+}^{2}e^{ix\sqrt{\kappa \Delta \varphi }/2-x\sqrt{\kappa \Delta \varphi }/2}+A_{2}\epsilon_{-}^{2}e^{ix\sqrt{\kappa \Delta \varphi }/2+x\sqrt{\kappa \Delta \varphi }/2}. \end{array} \end{array}$$

The saturable gain in the lasing cavity will act such that gain and decay terms are eliminated, leaving,

(A10) $$\begin{array}{c} \begin{array}{cc} {\tilde{E}}_{1} & =\left(A_{1}\epsilon_{+}^{1}+A_{2}\epsilon_{-}^{1}\right)e^{ix\sqrt{\kappa \Delta \varphi }/2}\\ {\tilde{E}}_{2} & =\left(A_{1}\epsilon_{+}^{2}+A_{2}\epsilon_{-}^{2}\right)e^{ix\sqrt{\kappa \Delta \varphi }/2}. \end{array} \end{array}$$

This shows explicitly that under the circumstances of operating near this Exceptional Point, the two counterpropagating electric fields oscillate at the same frequency of $\sqrt{\kappa \Delta \varphi }/\left(4\pi \tau_{p}\right)$, where the denominator comes from the fact that *x* is a round-trip index. While no beat frequency can be extracted from this system, the absolute frequency of each of the fields still corresponds to the applied perturbation, Δφ. Thus, there are ways that this could be used as a detector as described in [17]. However, because the modes by definition are non-orthogonal in the area surrounding this Exceptional Point, the issue of added noise remains. Another point of concern is that a lasing cavity requires a saturable gain which means that maintaining the two relationships, ($\alpha_{1}-\alpha_{2}{)}^{2}=4{\left|\kappa \right|}^{2}$, and $\alpha_{1}+\alpha_{2}=0$, that are required to place the system at the Exceptional Point becomes a challenge. Forcing the gain terms to be constants of $\alpha_{1}=\kappa =-\alpha_{2}$ results in the real electric fields tending to positive and negative infinity. This is also the case when one chooses the saturable terms,

(A11) $$\alpha_{\pm }=\frac{\alpha_{0}}{1+W_{i}/W_{s}}\pm \kappa .$$

The only way to maintain steady-state lasing seems to be to choose a saturable gain term in one resonator and a constant loss of κ in the other,

(A12) $$\begin{array}{cc} \alpha_{1} & =\frac{\alpha_{0}}{1+W_{i}/W_{s}} \end{array}$$

(A13) $$\begin{array}{cc} \alpha_{2} & =-\kappa . \end{array}$$

However, as Δφ is increased, the energy is unequally focused into the first resonator such that $\alpha_{1}$ decreases. This means, then, that the only time that the EP relationship is maintained is at Δφ=0.

## References

[1] Fabry C., Pérot A. (1897). Sur les franges des lames minces argentées et leur application la mesure de petites epaisseurs d’air. Ann. Chim. Phys..

[2] Michelson A.A. (1918). On the Correction of Optical Surfaces. Proc. Natl. Acad. Sci. USA.

[3] Sagnac M.G. (1913). L’éther lumineux démontré par l’effet du vent relatif d’éther dans un interféromètre en rotation uniforme. C. R. Acad. Sci..

[4] Sagnac M.G. (1913). Sur la preuve de la réalité de l’éther lumineux démontré par l’expérience de l’interférographe tournant. C. R. Acad. Sci..

[5] Born M., Wolf E. (1980). Principles of Optics—Electromagnetic Theory of Propagation, Interference and Diffraction of Light.

[6] Aronowitz F., Ross M. (1971). The laser gyro. Laser Applications.

[7] Arissian L., Diels J.C. (2014). Intracavity phase interferometry: Frequency comb sensors inside a laser cavity. Laser Photonics Rev..

[8] Krylov A.A., Chernykh D.S., Obraztsova E.D. (2018). Colliding-pulse hybridly mode-locked erbium-doped all-fiber soliton gyrolaser. Laser Phys..

[9] Zavadilová A., Vyhlidal D., Kubecek V., Sulc J. (2014). Subharmonic synchronously intracavity pumped picosecond optical parametric oscillator for intracavity phase interferometry. Laser Phys. Lett..

[10] Bender C.M., Boettcher S. (1998). Real Spectra in Non-Hermitian Hamiltonians Having *PT* Symmetry. Phys. Rev. Lett..

[11] El-Ganainy R., Makris K.G., Khajavikhan M., Musslimani Z.H., Rotter S., Christodoulides D.N. (2018). Non-Hermitian physics and PT symmetry. Nat. Phys..

[12] Wiersig J. (2020). Review of exceptional point-based sensors. Photon. Res..

[13] Smith D.D., Chang H. (2020). Excess noise: Why exceptional points do not increase sensor precision. arXiv.

[14] Wang H., Lai Y.H., Yuan Z., Suh M.G., Vahala K. (2020). Petermann-factor sensitivity limit near an exceptional point in a Brillouin ring laser gyroscopet. Nat. Commun..

[15] Egorov D.A., Olekhnovich R.O., Untilov A.A., Aleinik A.S., Deineka G.B., Strigalev V.E. (2011). Study on dead zones of fiber-optic gyros. Gyroscopy Navig..

[16] Horstman L., Hsu N., Hendrie J., Smith D., Diels J.C. (2020). Exceptional points and the ring laser gyroscope. Photon. Res..

[17] Smith D.D., Chang H., Horstman L., Diels J.C. (2019). Parity-time-symmetry-breaking gyroscopes: Lasing without gain and subthreshold regimes. Opt. Express.

[18] Smith D.D., Myneni K., Odutola J.A., Diels J.C. (2009). Enhanced sensitivity of a passive optical cavity by an intracavity dispersive medium. Phys. Rev. A.

[19] Smith D.D., Chang H., Myneni K., Rosenberger A.T. (2014). Fast-light enhancement of an optical cavity by polarization mode coupling. Phys. Rev. A.

[20] Pati G.S., Salit M., Salit K., Shahriar M.S. (2007). Demonstration of a Tunable-Bandwidth White-Light Interferometer Using Anomalous Dispersion in Atomic Vapor. Phys. Rev. Lett..

[21] Salit M., Pati G.S., Salit K., Shahriar M.S. (2007). Fast-light for astrophysics: Super sensitive gyroscopes and gravitational wave detectors. J. Mod. Opt..

[22] Shahriar M.S., Pati G.S., Tripathi R., Gopal V., Messall M., Salit K. (2007). Ultrahigh enhancement in absolute and relative rotation sensing using fast and slow light. Phys. Rev. A.

[23] Petermann K. (1979). Calculated spontaneous emission factor for double-heterostructure injection lasers with gain-induced waveguiding. IEEE J. Quantum Electron..

[24] Hall J., Ye J. (2003). Optical frequency standards and measurement. IEEE Trans. Instrum. Meas..

[25] Reichert J., Holzwarth R., Udem T., Hänsch T.W. (1999). Measuring the frequency of light with mode-locked lasers. Opt. Commun..

[26] Udem T., Reichert J., Holzwarth R., Hänsch T. (1999). Absolute optical frequency measurement of the cesium *D*_1_ line with a mode-locked laser. Phys. Rev. Lett..

[27] Udem T., Reichert J., Holzwarth R., Hänsch T. (1999). Accurate measurement of large optical frequency differences with a mode-locked laser. Opt. Lett..

[28] Jones R.J., Diels J.C., Jasapara J., Rudolph W. (2000). Stabilization of the frequency, phase, and repetition rate of an ultra-short pulse train to a Fabry-Perot reference cavity. Opt. Commun..

[29] Arissian L., Diels J.C. (2009). Investigation of Carrier to Envelope Phase and repetition rate—Fingerprints of mode-locked laser cavities. J. Phys. B At. Mol. Opt. Phys..

[30] Arissian L., Diels J.C. (2007). Carrier to envelope and dispersion control in a cavity with prism pairs. Phys. Rev. A.

[31] Masuda K., Hendrie J., Diels J.C., Arissian L. (2016). Envelope, Group and Phase velocities in a nested frequency comb. J. Phys. B.

[32] Coddington I., Newbury N., Swann W. (2016). Dual-comb spectroscopy. Optica.

[33] Muraviev A., Konnov D., Vodopyanov K. (2020). Broadband high-resolution molecular spectroscopy with interleaved mid-infrared frequency combs. Sci. Rep..

[34] Velten A., Schmitt-Sody A., Diels J.C. (2010). Precise intracavity phase measurement in an optical parametric oscillator with two pulses per cavity round-trip. Opt. Lett..

[35] Diels J.C., Horstman L., Hsu N., Hendrie J. (2021). Limits of Resolution for Sensors based on Correlated Frequency Combst. CLEO: 2021.

[36] Hsu N. (2020). Ultrashort Pulses and Frequency Combs: Characterizations, Manipulations, and Applications. Ph.D. Thesis.

[37] Diddams S., Atherton B., Diels J.C. (1996). Frequency locking and unlocking in a femtosecond ring laser with the application to intracavity phase measurements. Appl. Phys. B.

[38] Dennis M.L., Diels J.C., Lai M. (1991). The femtosecond ring dye laser: A potential new laser gyro. Opt. Lett..

[39] Lai M., Diels J.C., Dennis M. (1992). Nonreciprocal measurements in fs ring lasers. Opt. Lett..

[40] Schmitt-Sody A., Velten A., Masuda K., Diels J.C. (2010). Intra-cavity mode locked Laser Magnetometer. Opt. Commun..

[41] Schwindt P.D.D., Linseth B., Knappe S., Shah V., Kitching J. (2007). Chip-scale atomic magnetometer with improved sensitivity by use of the Mx technique. Appl. Phys. Lett..

[42] Singh S.P. (2014). Magnetoencephalography: Basic principles. Ann. Indian Acad. Neurol..

[43] Bohn M.J., Diels J.C., Jain R.K. (1997). Measuring Intracavity Phase Changes Using Double Pulses in a Linear Cavity. Opt. Lett..

[44] Quintero-Torres R., Ackerman M., Navarro M., Diels J.C. (2004). Scatterometer using a bidirectional ring laser. Opt. Commun..

[45] Navarro M., Chalus O., Diels J.C. (2006). Mode-locked ring lasers for backscattering measurement of mirror. Opt. Lett..

[46] Diddams S., Diels J.C., Atherton B. (1998). Differential intracavity phase spectroscopy of a three-level system in samarium. Phys. Rev. A.

[47] Horstman L. (2021). Intracavity Phase Interferometry Based Fiber Sensors. Ph.D. Thesis.

[48] Tang X., Tian J., Zhao J., Jin Z., Liu Y., Liu J., Chen T., Li J. (2021). Structure design and optimization of SOI high-temperature pressure sensor chip. Microelectron. J..

[49] Malykin G.B. (2000). The Sagnac effect: Correct and incorrect explanations. Physics-Uspekhi.

[50] Forshaw J.R., Smith A.G. (2009). Dynamics and Relativity.

[51] Ren J., Hodaei H., Harari G., Hassan A.U., Chow W., Soltani M., Christodoulides D., Khajavikhan M. (2017). Ultrasensitive micro-scale parity-time-symmetric ring laser gyroscope. Opt. Lett..

[52] Siegman A.E. (1989). Excess spontaneous emission in non-Hermitian optical systems. II. Laser oscillators. Phys. Rev. A.

[53] Hamel W.A., Woerdman J.P. (1990). Observation of enhanced fundamental linewidth of a laser due to nonorthogonality of its longitudinal eigenmodes. Phys. Rev. Lett..

[54] Van der Lee A.M., van Druten N.J., Mieremet A.L., van Eijkelenborg M.A., Lindberg A.M., van Exter M.P., Woerdman J.P. (1997). Excess Quantum Noise due to Nonorthogonal Polarization Modes. Phys. Rev. Lett..

[55] Chekalin S.V., Kryukov P.G., Matveetz Y.A., Shatherashvili O.B. (1974). The processes of formation of ultrashort laser pulses. Opto-Electronics.

[56] Diels J.C., Rudolph W. (2006). Ultrashort Laser Pulse Phenomena.

[57] Chow W.W., Gea-Banacloche J., Pedrotti L.M., Sanders V.E., Schleich W., Scully M.O. (1985). The ring laser gyro. Rev. Mod. Phys..

[58] Aronowitz F., Collins R.J. (1966). Mode coupling due to backscattering in a He-Ne traveling-wave ring laser. Appl. Phys. Lett..

[59] Aronowitz F., Collins R.J. (1970). Lock-in and intensity-phase interaction in the ring laser. J. Appl. Phys..

[60] Schmitt-Sody A., Arissian L., Velten A., Diels J.C., Smith D. (2008). Rabi cycling of two pulses in a mode-locked ring laser cavity with electro-optical control. Phys. Rev. A.

[61] New G. (1995). The Origin of Excess Noise. J. Mod. Opt..

[62] Hendrie J., Lenzner M., Akhamiardakani H., Diels J.C., Arissian L. (2016). Impact of resonant dispersion on the sensitivity of intracavity phase interferometry and laser gyros. Opt. Express.

[63] Engen A.V., Diddams S., Clement T.S. (1998). Dispersion measurements of water using white light interferometry. Appl. Opt..

